# Reactivation of dormant anti-tumor immunity – a clinical perspective of therapeutic immune checkpoint modulation

**DOI:** 10.1186/s12964-016-0155-9

**Published:** 2017-01-19

**Authors:** Richard Greil, Evelyn Hutterer, Tanja Nicole Hartmann, Lisa Pleyer

**Affiliations:** 1 Third Medical Department with Hematology, Medical Oncology, Hemostaseology, Infectious Disease and Rheumatology, Oncologic Center, Paracelsus Medical University Salzburg, Müllner Hauptstraße 48, A-5020 Salzburg, Austria; 2 Salzburg Cancer Research Institute (SCRI) - Laboratory for Immunological and Molecular Cancer Research (LIMCR), Salzburg, Austria; 3 Arbeitsgemeinschaft Medikamentöse Tumortherapie (AGMT) Study Group, Salzburg, Austria; 4 Cancer Cluster Salzburg (CCS), Salzburg, Austria

**Keywords:** Checkpoint inhibitor, Cancer, Immunoediting, Exhaustion, Mutational load, T cell repertoire

## Abstract

In favor of their outgrowth, cancer cells must resist immune surveillance and edit the immune response. Cancer immunoediting is characterized by fundamental changes in the cellular composition and the inflammatory cytokine profiles in the microenvironment of the primary tumor and metastatic niches, with an ever increasing complexity of interactions between tumor cells and the immune system. Recent data suggest that genetic instability and immunoediting are not necessarily disparate processes. Increasing mutational load may be associated with multiple neoepitopes expressed by the tumor cells and thus increased chances for the immune system to recognize and combat these cells. At the same time the immune system is more and more suppressed and exhausted by this process. Consequently, immune checkpoint modulation may have the potential to be most successful in genetically highly altered and usually extremely unfavorable types of cancer. Moreover, the fact that epitopes recognized by the immune system are preferentially encoded by passenger gene mutations opens windows of synergy in targeting cancer-specific signaling pathways by small molecules simultaneously with antibodies modifying T-cell activation or exhaustion.

This review covers some aspects of the current understanding of the immunological basis necessary to understand the rapidly developing therapeutic endeavours in cancer treatment, the clinical achievements made, and raises some burning questions for translational research in this field.

## Background

Tumor immunotherapy has a long-standing history. Starting with the work of William Coley in the 1890s [1], some progress in the treatment of malignancies was achieved with the introduction of interferons, interleukin-2 given either systemically or used for in vitro expansion of T-cells and reinfusion of lymphokine-activated killer cells. At the price of substantial side effects, success was seen in hematological cancers such as multiple myeloma, follicular lymphoma and myeloproliferative disorders, including chronic myeloid leukemia and polycythemia vera (interferons) and acute myeloid leukemia (AML) post allogeneic stem cell transplantation (interleukin-2) [2, 3]. In solid cancers, including melanoma and renal cell cancer, some long-term survivors and even cures were observed with extremely high-dose immuno- or chemoimmunotherapy approaches with interferons or interleukin-2 in the metastatic setting, but toxicities were severe [4, 5].

Substantial progress has later been made with the introduction of monoclonal antibodies (MAb) inducing apoptosis and/or eliciting antibody- or complement-dependent cytotoxicity after binding to tumor antigens. Just to name a few, anti-CD20- [6], anti-Her2- [7], anti-epidermal growth factor receptor (EGFR)- [8] and anti-CD38-MAb [9] are highly efficient in the clinics. Antibodies armed with toxins (*eg* brentuximab vedotin [10], gemtuzumab ozogamicin [11], trastuzumab emtansine [12], rovalpituzumab tesirine [13], denileukin diftitox [14]) have also proven to be successful.

The most exciting recent progress in the treatment of cancers, however, is derived from the better understanding of how tumor cells escape immune recognition [15] and how they exhaust, suppress and even kill immunocompetent T-cells directed against the tumor [16–20]. T-cell exhaustion is thereby induced by consistent antigen exposure leading to altered T-cell differentiation, loss of effector functions and anergy as well as upregulation and coexpression of inhibitory receptors that are also used as exhaustion markers (*eg* programmed death 1 (PD1)), and alterations of other key characteristics (for reviews see [21–23]). In addition, cancer cells cleverly expand regulatory T-cells (Tregs) [24] and further B-, natural killer- and dendritic-regulatory cells (for review see [25]) in order to prime the microenvironment towards a tumor supportive milieu. Under normal conditions, immune checkpoint molecules serve to regulate T-cell responses, which is necessary to avoid uncontrolled expansion resulting in organ destruction and fatal outcomes. Tumor cells use these intrinsic ‘brakes’ of the immune system as immune escape mechanisms by inducing functionally exhausted T-cells [15, 25].

The generality of these mechanisms across most -if not all- cancer types implies a yet unexploited applicability of drugs targeting immune suppression in a wide range of tumor entities. In fact, antibodies counteracting suppression of the T-cell receptor (TCR) signaling *via* CD28/cytotoxic T-lymphocyte-associated protein 4 (CTLA-4) (*eg* ipilimumab), or interfering with T-cell exhaustion *via* the PD1/PD ligand 1 (PDL1) axis (*eg* nivolumab, pembrolizumab, atezolizumab, durvalumab etc.) display impressive therapeutic efficacy in melanoma [26–32], squamous [33] and non-squamous non-small-cell lung cancer (NSCLC) [34], squamous cell cancer of the head and neck [35], renal [36], urothelial cancers [37] and Hodgkin’s disase [38, 39]. Anticancer drugs targeting these so-called ‘immune checkpoints’ on T-cells have been termed ‘checkpoint inhibitors’. The opposite side of the coin, however, is the relevant side effect profile of checkpoint inhibitors, with some patients developing autoimmunity against various organs including hypophysis, adrenal glands, beta cells of the pancreas, thyroid, lungs, liver, gut and nerves. In fact, knockout of PD1 [40] or CTLA-4 [41] resulted in severe and lethal autoimmune diseases in murine models. In humans, treatment with anti-CTLA-4 or anti-PD1 MAbs led to significant autoimmune phenomena and the number of patients with treatment-related grade 3–4 side effects increased up to 55% when both drugs were combined [26].

**Fig. 1:**
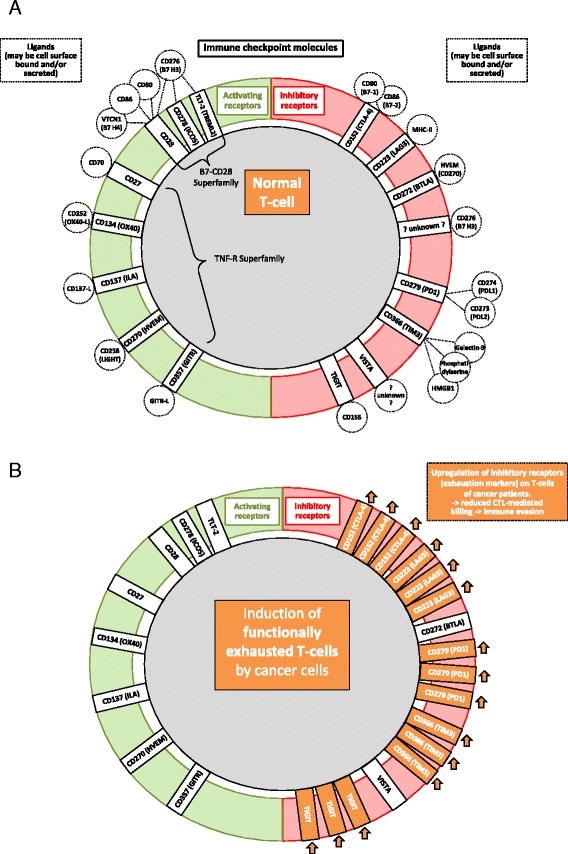
Checkpoint receptors on T cells. Figure 1 **a** shows negative checkpoint receptors (NCRs) on the right, and activating (costimulatory) checkpoint receptors (ACRs) of a normal T-cell on the left. The balance between the expression of these receptors, and the ligation with respective ligands, determines the functional status of the T-cell during varying physiological processes. Figure 1 **b** schematically shows how cancer cells may modulate T-cells, to prefentretially express and upregulate NCRs. Therefore, T-cells of cancer patients often become exhausted, anergic, and/or incapable of efficiently attacking and killing the cancer cells. This is one of the mechanisms by which the malignant cells induce tumor imune escape

There is need to systematically clarify the potential exploitation of targeting individual receptors expressed by T-cells, with the aim of circumventing the immunosuppressive effects cleverly used by cancer cells to evade host anti-tumor immune responses. In brief, T-cells exhibit various activating and inhibitory ‘checkpoint’ receptors or molecules (Fig. 1a).

Activating costimulatory immune checkpoint molecules expressed by T-cells include (i) the B7-CD28 superfamily, which encompasses CD28 (the receptor for CD80 and CD86), CD278 (inducible T-cell costimulator (ICOS) and TREML-2/TLT-2 (Trem-like transcript 2 protein), and (ii) members of the tumor necrosis factor receptor (TNFR) superfamily such as CD27, CD134 (OX40), CD137 (induced by lymphocyte activation (ILA)), CD270 (herpesvirus entry mediator (HVEM)) and CD357 (glucocorticoid-induced TNFR family related gene (GITR)) (reviewed *eg* in [42]).

Inhibitory checkpoint molecules found on T-cells include CD152 (CTLA-4), CD223 (lymphocyte activation gene 3 (LAG3)), CD272 (B- and T-lymphocyte attenuator (BTLA)), CD279 (PD1) and CD366 (T-cell immunoglobulin and mucin protein 3 (TIM3)), V-domain Ig suppressor of T-cell activation (VISTA), as well as the newly discovered T-cell immunoreceptor with Ig and ITIM domains (TIGIT).

These checkpoint molecules are extremely important, as they help the body to discriminate between ‘foreign’ and ‘self’ as well as help restrain immune responses against foreign targets, while sparing ‘self’. They are often deregulated in cancer, *eg* by expression or upregulation of inhibitory molecules by the cancer cells themselves, and/or by production of soluble factors by the cancer cells that result in downregulation or blockage of activating receptors, or in ligation and/or upregulation of inhibitory receptors on T-cells, respectively (Fig. 1b). In addition, the phenomenon of T-cell exhaustion can be induced by cancer, thereby hindering anti-tumor immune control (reviewed in [21, 22]).

Numerous drugs have been developed to intercept the malignant control of the immune system by specifically targeting these checkpoint molecules on T-cells. Activating checkpoint molecules can be therapeutically targeted with agonistic molecules, whereas inhibitory immune checkpoint molecules expressed by T-cells can be targeted with blocking antibodies, and the respective drugs that are currently tested and/or have been approved are listed in Tables 1, 2, 3 and 4.

**Table 1:** Current status of agents targeting negative checkpoint receptors (NCRs)

Drug Target, (Synonym)	Effect on T-cells and immune system	Compound (Synonym)	Drug company (Trade name)	Drug type (application)	Status	Indication
CD152 (CTLA4)	Blocking of CTLA4, activation of T-cells and anti-tumor immune response	Ipilimumab (MDX-010)	BMS (Yervoy)	Human MAb, IgG1ĸ (i.v., Q3W)	FDA 25.03.2011 EMA 25.07.2011 Investigational	MEL (metastatic and adjuvant) MEL NSCLC, SCLC
		Tremelimumab (CP-675,206)	AstraZeneca	Human MAb, IgG2ĸ (i.v., Q4-12 W)	Phase II	Mesothelioma, MEL
CD223 (LAG3)	Blocking of LAG3-mediated immune down-regulation; Activation and expansion of tumor antigen-specific T-effector cells; activation of antigen presenting cells to remove tumor debris;	BMS-986016	BMS	MAb, (i.v.)	Phase I	Relapsed hematologic malignancies
		LAG-525	Novartis	Humanized MAb, (i.v.)	Phase I	Advanced solid tumors
		IMP-701	Novartis	MAb, (i.v.)	Preclinical	No data available; Cancer
		IMP-321	Prima BioMed	Recombinant human soluble LAG3 MAb fragment (fusion protein), (i.v.)	Phase I/II	Pancreatic cancer, MEL, MRCC, metastatic breast cancer
CD223 (LAG3)	Blocks LAG3; Depletion of activated autoaggressive T-cells	GSK-2831781 (initially IMP-731)	GSK	Humanized MAb, (i.v.)	Phase I	Psoriasis, autoimmune diseases
CD272 (BTLA)	-	-	-	-	Preclinical	In vitro, murine models
CD279 (PD1)	Blocking of PD1; Activation of anti-tumor immune response *via* prevention of CD8+ T-cell exhaustion	Nivolumab (BMS-936558) (MDX-1106)	BMS (Opdivo)	Human MAb, IgG4ĸ (i.v.)	FDA 22.12.2014 EMA 16.07.2015	MEL, NSCLC, RCC, M. Hodgkin MEL, NSCLC, RCC
		Pembrolizumab (Lambrolizumab) (MK-3475)	Merck (Keytruda)	Humanized MAb, IgG4ĸ (i.v., Q3W)	FDA 04.09.2015 EMA 30.07.2015	MEL, NSCLC MEL
		BGB-A317	BeiGene	Humanized MAb, (i.v. Q2-3 W)	Phase I	Advanced solid tumors, B-cell malignancies
		TSR-042	Tesaro, AnaptysBio	Humanized MAb, (i.v.)	Phase I	Advanced or metastatic solid tumors
		PDR-001	Novartis	Humanized MAb IgG4ĸ, (i.v.)	Phase I/II	Advanced malignancies, rec./metastatic nasopharyngeal carcinoma
		Pidilizumab (CT-011) (MDV9300)	Medivation	Humanized MAb IgG1ĸ, (i.v.)	Phase II	DLBCL, relapsed myeloma, follicular lymphoma
		AMP-224 (AFC-111CL)	Creative Biolabs	ADCC enhanced MAb. (i.v.)	Phase I	Advanced, refractory cancer, metastatic CRC
		MEDI-0680 (AMP-514)	MedImmune	Humanized MAb IgG4ĸ, (i.v.)	Phase I	Advanced solid tumors
CD366 (TIM3)	Blocking of TIM3; Activation of anti-tumor immune response *via* prevention of CD8+ T-cell exhaustion	TSR-022	Tesaro, AnaptysBio	Humanized MAb IgG4, (i.v.)	Phase I	Advanced solid tumors
		MBG-453	Novartis	MAb, (i.v.)	Phase I	Advanced malignancies
VISTA CD274 (PDL1)	Blocking of VISTA and PDL1; Activation of T-cell proliferation & cytokine production	CA-170 (AUPM-170)	Curis, Aurigene	Small molecule antagonist (p.o.)	Phase I	Advanced solid tumors or lymphomas, nonresponsive to available therapies
TIGIT	-	-	-	-	Preclinical	Murine cancer models

*MAb *indicates monoclonal antibody, *i.v.* intravenous, *p.o.* per os, *FDA* Agency for Food and Drug Administraton, *EMA* European Medicines Agency, *MEL* metastatic melanoma, *NSCLC* non small cell lung cancer, *SCLC* small cell lung cancer, *MRCC* metastatic renal cell carcinoma, *DLBCL* diffuse large B-cell lymphoma, *CRC* colorectal cancer

**Table 2:** Current status of therapeutic agents targeting ligands of negative checkpoint receptor (NCR)

Drug Target, (Synonym)	Effect on T-cells and immune system	Compound (Synonym)	Drug company (Trade name)	Drug type (application)	Status	Indication
CD270 (HVEM)	Activation/Inhibition	-	-	-	Preclinical	-
CD274 (PDL1)	Blocks PD1/PDL1 ligation; Activation of anti-tumor immune response *via* prevention of CD8+ T-cell exhaustion	Durvalumab (MEDI-4736)	AstraZeneca	Human MAb IgG1ĸ (i.v., Q2-3 W)	FDA 17.02.2016 BTD Phase III Phase I/II	Metastatic urothelial cancer NSCLC Head and neck, gastric cancer, pancreatic cancer, hematologic malignancies, myelodysplastic syndromes
		Atezolizumab (MPDL-3280A) (RG7446)	Roche Genentech (Tecentriq)	Humanized MAb IgG1ĸ, (i.v., Q3W)	FDA 18.05.2016 Phase III	Metastatic urothelial cancer NSCLC
		Avelumab (MSB-0010718C)	Merck & Pfizer	Human MAb IgG1ĸ, (i.v., Q2W)	Phase III	Advanced malignancies, bladder cancer, ovarian cancer, MRCC, head and neck, NSCLC, gastric cancer, Merkel cell carcinoma, M. Hodgkin
		BMS-936559 (MDX-505)	BMS	Human MAb IgG4 ĸ, (i.v., Q2W)	Phase I	MEL, advanced refractory solid tumors and hematologic malignancies
CD274 (PDL1) VISTA	Blocking of PDL1 and VISTA - > indirect activation of T-cell proliferation & cytokine production	CA-170 (AUPM-170)	Curis, Aurigene	Small molecule antagonist (p.o., BID Q4D)	Phase I	Advanced solid tumors or lymphomas, non-responsive to available therapies
HGMB1	Sensitizes MDR AML cells to chemotherapy, significant decrease in AML cell proliferation	miR181b	-	Inhibits HMGB1 expression	Preclinical	In vitro

*MAb *indicates monoclonal antibody, *i.v.* intravenous; *p.o﻿.﻿* per os,﻿ *FDA* Agency for Food and Drug Administraton, *BTD* Breakthrough Therapy Designation, *NSCLC* non small cell lung cancer, *MRCC* metastatic renal cell carcinoma, *MEL* metastatic melanoma, *p.o.* per os, *MDR* multi-drug resistant, *AML* Acute myeloid leukemia

**Table 3:** Current status of therapeutic agents targeting activating checkpoint receptors

Drug Target (Synonym)	Effect on T-cells and immune system	Compound (Synonym)	Drug company (Trade name)	Drug type (application)	Status	Indication
CD27	Binds and activates CD27 - > activation of anti-tumor immune response; Treg depletion; Direct targeting of CD27 expressing tumors	Varlilumab (CDX-1127)	Celldex	Human mAb IgG1ĸ, (i.v., D1,8,15,22 Q8W)	Phase I/II	CRC, NSCLC, RCC, MEL, ovarian cancer, head and neck squamous cell carcinoma
CD28	Immunosuppression *via* preferential activation of Tregs	TAB-08 (TGN-1412)	TheraMab (TeGenero)	Humanized MAb IgG4ĸ, (i.v., Q1W)	Phase I/II	Rheumatoid arthritis, Psoriasis, SLE
CD134 (OX40)	Binds and activates OX40 - > increases T-cell proliferation and cytokine secretion - > activation of dormant anti-tumor immune response + reduction and suppression of Tregs	PF-04518600 (PF-8600)	Pfizer	Human MAb IgG2, (i.v.)	Phase I	Neoplasms
		MOXR-0916	Genentech	Humanized MAb IgG1, (i.v.)	Phase I	Advanced or metastatic tumors
		MEDI-0562	MedImmune	Humanized MAb (i.v.)	Phase I	Advanced or metastatic tumors
		MEDI-6469	MedImmune	Humanized MAb (i.v.)	Phase I	Metastatic CRC, locoregionally advanced head and neck cancer
CD137 (ILA, 4-IBB)	Activation and expansion of T-cells and anti-tumor immune response	Urelumab (BMS-663513)	BMS	Human MAb IgG4ĸ (i.v.)	Phase I	Recurrent glioblastoma, advanced solid tumors, hematologic neoplasms
		Utomilumab (PF-05082566)	Pfizer	Human MAb IgG2ĸ (i.v.)	Phase I	Avanced Solid Tumors
CD270 (HVEM)	Activation/Inhibition	-	-	-	Preclinical	-
CD278 (ICOS)	Activation	-	-	-	Preclinical	-
CD357 (GITR)	Binds and activates GITR - > activates tumor tumor antigen specific T-effector cells, suppresses Tregs - > activates anti-tumor immune response	MK-4166	Merck	MAb, (i.v.)	Phase I	Advanced Solid Tumors
		INCAGN-01876	Agenus	MAb, (i.v.)	Phase I	Advanced or Metastatic Solid Tumors
		TRX-518	Leap Therapeutics	Humanized MAb, (i.v.)	Phase I	Advanced Solid Tumors, MEL
		GWN-323	Novartis	MAb, (i.v.)	Phase I/II	Advanced solid tumors, lymphomas
TLT-2, TREML	Activation	-	-	-	Preclinical	-

*MAb* indicates monoclonal antibody, *i.v.* intravenous, *CRC* colorectal cancer, *NSCLC* non small cell lung cancer, *RCC* metastatic renal cell carcinoma, *MEL* metastatic melanoma, *SLE* systemic lupus erythematodes

**Table 4:** Current status of therapeutic agents targeting ligands activating checkpoint receptors

Drug Target (Synonym)	Effect on T-cells and immune system	Compound (Synonym)	Compound (Trade name)	Drug type (application)	Status	Indication
CD80 (B7-1) CD86 (B7-2)	T-cell costimulation blocker, inhibits T-cell proliferation and production of cytokines	Belatacept (LEA29Y)	BMS (Nulojix)	CTLA4 fusion-Ig, costimulation blocker	FDA approval 15.11.2011 EMA approval 07.07.2011	Prophylaxis of renal graft rejection Prophylaxis of renal graft rejection
		Abatacept	BMS (Orencia)	CTLA4 fusion-Ig, costimulation blocker	FDA approval 31.07.2011 EMA approval 25.01.2010	Rhematoid arthritis; Polyarticular juvenile idiopathic arthritis
CD80	Activates ADCC on B-NHL cells with upregulated CD80	Galiximab (IDEC-114)	Biogen	Humanized MAb IgG1, (i.v.)	Phase I/II Phase II	Untreated follicular lymphoma Relapsed/refractory M. Hodgkin
CD80 DNMT1	Upregulation of CD80 on cancer cells - > costimulatory activation of T-cells	Decitabine	Janssen-Cilag (Dacogen)	Deoxycytidine analogue, i.v. (D1-5, Q6W)	FDA approval 02.05.2006 EMA approval 28.09.2012	Myelodysplastic syndromes Acute myeloid leukemia
CD86 HDAC	Upregulation of CD86 on cancer cells, HDAC-I - > costimulatory activation of T-cells	Romidepsin (FR901228)	Gloucester Pharmaceuticals, Celgene (Istodax)	Depsipeptide, (i.v. Q1W)	FDA approval 05.11.2009 EMA refused approval 09.07.2012	Cutaneous T-NHL, PTCL
CD137-L	Induction of differentiation of AML cell samples in vitro	-	-	Recombinant CD137-L protein	Preclinical	In vitro
CD252 (OX40-L)	Binds and activates Ox40 by mimicking Ox40-L - > proliferation of TAA specific T-effector cells - > activation of anti-tumor immune response	MEDI-6383	MedImmune	Fusion protein	Phase I	Advanced solid tumors
CD252 (OX40-L)	Binds and inhibits Ox40L and the interaction with Ox40 - > inhibition of allergen induced immune response	Oxelumab (huMAb OX40L) (R4930)	Genentech	Human MAb IgG1, (i.v.)	Phase II	Mild allergic asthma
CD276 (B7-H3)	Inhibits CD276	Enoblituzumab (MGA-271)	MacroGenics	Humanized MAb IgG1ĸ, (i.v.)	Phase I	B7-H3 expressing refractory solid tumors
CD276 (B7-H3) CD3	Redirection of T-cells to kill B7-H3 overexpressing tumor cells	MGD-009	MacroGenics	Dual affinity retargeting protein	Phase I	B7-H3 expressing unresectable or metastatic solid tumors

*MAb* indicates monoclonal antibody, *i.v.* intravenous, *FDA* Agency for Food and Drug Administraton, *EMA* European Medicines Agency, *ADCC* antibody dependent cellular cytotoxicity, *NHL* non-Hodgkin lymphoma, *PTCL* peripheral T-cell lymphoma, *TAA* tumor associated antigen

## Predicting response to checkpoint blockade

The degree of efficacy of checkpoint inhibitors is highly divergent between different tumor types. This phenomenon may be attributed to differences in PDL1 expression on neoplastic or microenvironmental cells, suggesting that this marker should be quantified ahead of therapy. However, reported thresholds for PDL1 expression to predict the probability of response towards anti-PD1 MAbs vary between ≤ vs. >1% (for nivolumab) or ≤ vs. >50% (for pembrolizumab) and ≤ vs. >1%, 5% or 10% (for atezolizumab) with many reasons suggested, but none being really convincing in explaining these differences [43, 44]. Given the relevant side effect profiles of immune checkpoint inhibitors and their exceptionally high costs, novel and better predictors of response are therefore needed.

Notably, the mutational burden defined as the number of mutations per megabase, may correlate with -and thus predict the occurrence of- tumor-specific (neo)antigens (TSA) which are expressed on the tumor cell surface and presented to T-cells. T-cells exposed to TSA can learn to specifically target and eliminate (*ie* kill) tumor cells. In contrast to tumor-associated antigens (TAA), which are in essence massively overexpressed ‘normal antigens’ that also occur on normal, non-cancerous tissues of the body, TSA are true neoantigens that cannot be found on any non-malignant cell. TAA are much more common than TSA, and strategies targeting TAA molcules include *eg* the clinically widely successful targeting of CD20 in lymphomas and CD33 in AML, as briefly mentioned above. Targeting of TSA would in theory eliminate the bystander killing of normal cells, which also bear these molecules to a lesser extent (*eg* normal B-cells or myeloid cells for the two molecules mentioned above).

The mutational burden varies substantially over a range of 3–4 logs in different tumor entities [45] and even within the same tumor considerable interpatient variability may be observed. Provided that in tumors with high neoantigen frequency T cells are more prone to recognize TSA, but are exhausted by specific ligands during immunoediting, strategies aimed at re-instating T cell functions could be particularly effective in these patients [23]. This view is supported by the following facts:

- The response and efficacy of checkpoint inhibitors seems to be highest in tumor types with the highest mutational burden (*eg* melanoma, NSCLC) [46]. This is especially relevant in the light of the fact that patients with high numbers of mutations are usually weakly responsive to chemotherapy and/or rapidly develop chemo-resistance.
- In NSCLC patients treated with the PD1-inhibitor pembrolizumab, progression-free survival massively differed according to mutational burden in an as yet unseen manner [47]. This has also been observed in urothelial cancers treated with the anti-PDL1 antibody atezolizumab [48].
- Most patients with colon cancer usually do not respond to checkpoint inhibitors [49], however, in a small subset of advanced colorectal cancer patients high microsatellite instability due to deficient DNA mismatch repair [50] was observed, the occurrence of which has been associated with a high number of mutations, potentially resulting in an elevated expression of TSA on the tumor cell surface [51]. Treatment of these patients with PD1-inhibitors resulted in a response rate of nearly 40%, as compared to only 11% stable disease in those with microsatellite stability [51].

### APOBEC family members, mutational burden, the role of the immune system and its use as a predictor of response to checkpoint inhibitors

On average, 2–4 oncogenic driver gene mutations are present in various tumors [52, 53]. It is not clear yet whether the extent of driver gene mutations correlates with overall mutational burden (including passenger mutations) and how this impacts on checkpoint molecule expression on T-cells.

The AID (activation-induced cytidine deaminase)/APOBEC (apolipoprotein B mRNA editing enzyme catalytic polypeptide-like) gene family members are cytidine deaminases causing alterations in DNA and mRNA sequences by cytidine-to-uracil (C-U) transitions -with subsequent conversion of U to thymine (T) during DNA replication- a process called DNA/mRNA-editing that results in protection from parasitic viruses as well as protein and antibody diversity. Loss of cellular control of APOBEC activities results in DNA hypermutations, promiscuous RNA editing, and ultimately genetic instability and tumorigenesis (for recent review see [54, 55]). One of the main functions of AID is to regulate mutations in immunoglobulin (Ig) heavy and light chain genes during B-cell development in lymph nodes, thereby creating antibody diversity. AID also alters gene regulation by interfering with epigenetic DNA modification. However, AID is to a certain degree ‘leaky’ and may induce off-target gene mutations and/or translocations of oncogenes towards Ig genes, thereby promoting leukemogenesis and/or lymphomagenesis [56–58]. Similarly, other APOBEC family members, whose canonical function is to induce showers of mutations in cDNA intermediates of RNA viruses, contribute to tumor induction and progression in many types of neoplasias, including -but not limited to- chronic lymphocytic leukemia (CLL) and breast cancer [57–61]. Various members of the AID/APOBEC family may differ up to tenfold in their mutagenic capacity [62] and APOBEC enzymes may significantly drive subclonal evolution and tumor heterogeneity [63]. It is therefore not surprising that APOBEC family mutational signatures characterize patients with poor prognosis (*eg* in multiple myeloma), mostly *via* their involvement in generating translocations, which are often associated with adverse outcome [64]. In breast cancer, APOBEC-3B expression is associated with unfavorable clinicopathological features and poor outcome [65]. In line with these observations, APOBEC-3B expression has been associated with mutations of p53, as well as of the catalytic subunit of phosphatidylinositol 3-kinase [66].

On the other hand, activated members of the APOBEC family might increase the number of neoantigens, cancer-specific T-cell clones and may induce a broader TCR repertoire. Thus, APOBEC family member expression, function, or mutational pattern could serve as a biomarker for the response to checkpoint inhibitors and other immunomodulatory drugs. Initial evidence supporting this hypothesis includes:

- In non-invasive early urothelial cancers APOBEC-related mutational signatures were predominantly seen in high-risk tumors [67].
- In another small series of urothelial tumors, the expression of certain APOBEC family members (A3A, A3D and A3H) was associated with PDL1 positive mononuclear cells infiltrating the tumor and increased expression of the variants A3F_a and A3F_b correlated with upregulated expression of PDL1 on tumor cells [68], indicating that PDL1 may serve as a therapeutic target. As a side note, increased expression of A3D and A3H was associated with a better overall survival (OS) in this study, which seems paradoxical, or at least cannot be explained yet. Thus, further investigations concerning APOBEC expression patterns and response to checkpoint inhibitors are warranted.
- In high grade serous ovarian carcinomas APOBEC3 expression was significantly associated with T-cell infiltration and -seemingly paradoxically- with improved clinical outcome [69].
- Furthermore, breast cancer developed more commonly in women with germline APOBEC3B (A3B) deleting polymorphisms, but these women were not subject to unfavorable risk profiles or worse outcome [65], suggesting potential value for the determination of A3B deletion status in predicting response to checkpoint inhibitors.

All of the above data encourage deeper analyses of the correlation (and presumed interaction) between (i) APOBEC family member expression profiles, splice variants and/or polymorphisms and (ii) mutational burden, clonal evolution, and effects on expression profiles of immunomodulatory molecules and their function. This might lead to a better understanding and fine-tuning of immunotherapies in cancer.

### TCR repertoire and T-cell diversity in predicting response to immunotherapy

Often, driver gene mutations and associated atypical proteins remain immunologically silent. In fact, over 90% of cancer cell mutations recognized by CD4+ and CD8+ T-cells occur in passenger genes [45]. This suggests that the TCR repertoire broadens with increasing numbers of (passenger) gene mutations, resulting in a broader pool of T-cell clones capable of fighting cancer cells. This might be exploited with therapeutic strategies aimed at reactivating or boosting the host anti-tumor immune response. Therefore, although the presence of high mutational burden is generally acknowledged to be an adverse predictor of outcome across all tumor entitites, it may predict TCR diversity and thus good response to checkpoint inhibitors and/or activating immunotherapies.

Indeed, TCR diversity was associated with good clinical outcomes following treatment with the MAb ipilimumab targeting CTLA-4 in a small series of melanoma patients [70]. This was confirmed in conference papers by others, who show that a TCR diversity score higher than 20% is necessary for good outcome of melanoma patients receiving anti-CTLA-4 antibody treatment [71]. Interestingly, an inverse constellation was found for treatment with anti-PD1 antibodies [71]. However, as CTLA-4 blockade itself can broaden the TCR repertoire [72], this may partly explain the seeming discrepancies. Given the high number of immunomodulatory ligand/receptor pairs modifying cancer/T-cell interactions, a thorough investigation of these issues, ideally in prospective clinical trials, is warranted.

### Peripheral blood instead of tissue examinations of biomarkers

Most analyses of biomarkers thought to predict response to checkpoint inhibitors are currently performed in primary samples of tumor tissue. However, tumor biopsies are sometimes difficult or even impossible to obtain and, depending on the location of the tumor, may be associated with relevant side effects such as an increased risk of bleeding, organ perforation and/or infection, as well as high medical costs for the procedure itself. In addition, tissue biopsies usually cannot be performed continuously during the course of the disease due to these potential risks. Therefore, the actual status of tumor clone evolution and expansion or reduction of T-cell clones capable of actively combating the tumor, remains obscure in patients treated with checkpoint inhibitors. The provision of a rationale for therapeutic decision making and the choice of the optimal immunomodulatory drug most suitable to fight malignant subclones, would ideally require the serial analysis of (i) representative tumor DNA from tissue biopsies, and (ii) various T-cell subsets from peripheral blood.

Recently, the detection of circulating tumor DNA (ctDNA) has shown very high identification rates of mutations that were also found in primary tumors using a deep-coverage (15,000x) next-generation sequencing test of 70 genes [73].

Nevertheless, it has been shown that 9/10 gastrointestinal cancer patients had CD4+ and/or CD8+ tumor infiltrating lymphocytes that recognized 1–3 neo-epitopes from somatic mutations occurring in the respective cancers [74]. Moreover, very recently it has been demonstrated that circulating PD1+ lymphocytes from cancer patients were enriched in naturally occurring tumor-reactive and mutation-specific cells [75]. Exhaustion of T-cells has mostly, if not exclusively, been examined on tumor-infiltrating lymphocytes. Thus, longitudinal analyses and functional examination of T-cells during the natural disease course and/or during various treatment phases are lacking. In fact, it remains unknown at present, whether there is a correlation between ctDNA (*ie* type and frequency of mutations) and peripheral blood T-cell exhaustion profiles. This underlines the necessity to characterize peripheral blood T-cells within the frame of clinical trials that aim to aid the immune system to adapt to clonal tumor evolution *via* therapeutic immunoediting.

## Synergistic immunotherapeutic opportunities

### Interaction between various members of checkpoint inhibitors or immune-activators

The approach of repressing multiple pathways, or of combining repressive with immunostimulatory antibodies seems particulary exciting and is currently investigated in numerous trials (Table 5). In preclinical studies synergy for such approaches (*eg* inhibition of PD1 and activation of CD137, or combined inhibition of inhibitory checkpoint molecules) has been demonstrated [76–78]*.* Early phase I data support the view that such combinations of repressive with activating MAbs are feasible ([78]*;* NCT00803374, NCT02253992, NCT00351325). However, these therapeutic approaches must be viewed with caution and be closely monitored, given the overwhelming activation of autoimmunity which could arise.

**Table 5:** Clinical trials testing combined targeting strategies of more than 1 checkpoint receptor

Drug target (Synonym)	Compound (Synonym)	Status	Indication	ClinicalTrials.gov identifier
CD134 (OX40) CD137 (ILA, 4-IBB)	PF-04518600 Utomilumab (PF-05082566)	Phase I	Neoplasms	NCT02315066
CD134 (OX40) CD137 (ILA, 4-IBB) CD274 (PDL1)	PF-04518600 Utomilumab (PF-05082566) Avelumab (MSB-0010718C)	Phase I	Neoplasms	NCT02554812
CD137 (ILA, 4-IBB) CD279 (PD1)	PF-05082566 MK-3475	Phase I	Advanced solid tumors	NCT02179918
CD123 (OX40) CD274 (PDL1) VEGF	MOXR0916 Atezolizumab (MPDL-3280A) Bevacizumab	Phase I	Advanced or metastatic solid tumors	NCT02410512
CD134 (OX40) CD152 (CTLA4) CD274 (PDL1)	MEDI-0562 Tremelimumab (CP-675,206) Durvalumab (MEDI-4736)	Phase I	Advanced or metastatic solid tumors	NCT02705482
CD134 (OX40) CD152 (CTLA4) CD274 (PDL1)	MEDI-6469 Tremelimumab (CP-675,206) Durvalumab (MEDI-4736)	Phase I/II	Advanced solid tumors or DLBCL	NCT02205333
CD252 (OX40L) CD274 (PDL1)	MEDI-6383 Durvalumab (MEDI-4736)	Phase I	Recurrent or metastatic solid tumors	NCT02221960
CD276 (B7-H3) CD152 (CTLA4)	Enoblituzumab (MGA-271) Ipilimumab	Phase I	B7-H3 expressing solid tumors	NCT02381314
CD276 (B7-H3) CD279 (PD1)	Enoblituzumab (MGA-271) Pembrolizumab	Phase I	B7-H3 expressing solid tumors	NCT02475213
VISTA CD274 (PDL1)	CA-170 (AUPM-170)	Phase I	Advanced solid tumors or lymphomas, non-responsive to available therapies	NCT02812875
CD152 (CTLA4) CD274 (PDL1) Cytotoxic	Tremelimumab (CP-675,206) Durvalumab (MEDI-4736) Chemotherapy	Phase I Phase I Phase I	Resectable CRC with liver metastases Solid malignancies Multiple Myeloma	NCT02754856 NCT02537418 NCT02716805
CD152 (CTLA4) CD274 (PDL1) Cytotoxic	Tremelimumab (CP-675,206) Durvalumab (MEDI-4736) Radiotherapy	Phase I Phase II Phase II	Unresectable pancreatic cancer NSCLC, CRC Relapsed SCLC	NCT02311361 NCT02888743 NCT02701400
CD152 (CTLA4) CD274 (PDL1)	Tremelimumab (CP-675,206) Durvalumab (MEDI-4736)	Phase I Phase II Phase II Phase II Phase III	Advanced solid tumors Unresectable hepatocellular carcinoma Metastatic Her2 negative breast cancer Head and neck cancer NSCLC	NCT02261220 NCT02519348 NCT02536794 NCT02319044 NCT02453282
CD274 (PDL1) CD279 (PD1)	Durvalumab (MEDI-4736) MEDI-0680	Phase I/II	Advanced malignancies	NCT02118337
CD137 (ILA, 4-IBB) CD279 (PD1)	Urelumab Nivolumab (BMS-936558)	Phase I Phase II	Recurrent glioblastoma Cisplatin-ineligible bladder carcinoma	NCT02658981 NCT02845323
CD152 (CTLA4) CD137 (ILA, 4-IBB)	Ipilimumab BMS-663513	Phase I	Advanced melanoma	NCT00803374
CD223 (LAG3) CD279 (PD1)	BMS-986016 Nivolumab (BMS-936558)	Phase I	Relapsed hematologic malignancies	NCT02061761
CD366 (TIM3) CD279 (PD1)	MBG-453 PDR-001	Phase I/II	Advanced malignancies	NCT02608268
CD279 (PD1) CD357 (GITR)	PDR-001 GWN-323	Phase I/II	Advanced malignancies and lymphomas	NCT02740270
CD279 (PD1) CD223 (LAG3)	PDR-001 LAG-525	Phase I/II	Advanced malignancies	NCT02460224
CD357 (GITR) CD279 (PD1)	MK-4166 Pembrolizumab (MK-3475)	Phase I	Advanced solid tumors	NCT02132754

*DLBCL*indicates diffuse large B-cell lymphoma, *CRC* colorectal cancer, *NSCLC* non small cell lung cancer, *SCLC* small cell lung cancer, *MRCC* metastatic renal cell carcinoma

Data in melanoma have shown that nivolumab outcompetes ipilimumab and that the combination of both is superior over single treatment strategies [28]. In this regard it is interesting to note that in a murine model acquired resistance against anti-PD1 antibodies was accompanied by an upregulation of another exhaustion marker, TIM3. The resistance could be broken by inhibition of TIM3 with a blocking antibody and these preclinical mouse data were supported by in vivo findings in two lung cancer patients [79]. Therefore the combination -or sequential application- of *eg* anti-PD1 or anti-PDL1 antibodies with anti-TIM3 antibodies is an approach that should be further evaluated in controlled clinical trials.

Notably, TIM3 is expressed on tumor-infiltrating Tregs (CD4+, CD25+, Foxp3+), which suppress CD8+ cytotoxic T-cells (CTLs) [80]. Blocking of TIM3 would thus reduce the Treg mediated suppression of (tumor-specific) CTLs and allow them to target the tumor. However, the degree to which such an effect might be offset by TIM3 expression on CD4+ [81] and CD8+ [82] effector T cells remains to be determined, particularly as TIM3 expression was also associated with improved survival under certain conditions [83]. Clearly, a systematic serial analysis of changes in the expression profiles of immunodulatory molecules during immunoediting in carcinogenesis, progression of disease as well as during (effective) treatment needs to be carried out in individual tumor entities in order to dissect optimal time points and types of immunologic interventions.

## Synergistic opportunities with other therapies

### Off-target effects of small molecules on T-cells

Kinase inhibitors might synergize with immunotherapy in combating cancer, even without direct interaction of the molecular targets. In fact, phase I clinical trials have shown a synergy between gefitinib, which targets EGFR with the PD1 checkpoint inhibitor durvalumab in EGFR mutated NSCLC patients with ~80% response rates [84]*.* In addition, drugs targeting the proteins of mutated driver genes might directly increase the re-activation of the specific immune system exerted by checkpoint inhibitors. In part, these effects may be caused by interference of some kinase inhibitors with signaling pathways essential for T-cell function, activation, survival and proliferation. Indeed, it has recently been shown that the mitogen-activated protein kinase (MAPK) kinase (MEK) inhibitor cobimetinib increased major histocomaptibility complex (MHC) class I molecule expression on cancer cells and induced a 17% response rate in colorectal cancer patients treated with the anti-PDL1 mAb atezolizumab [85]. Midostaurin, an flt-3 inhibitor with a broad kinase inhibition dendrogram, increases OS in AML patients (when added to daunorubicin and cytarabine) [86], yet does not hamper TCR signaling or T-cell activation [87]. Its effect on expression and function of checkpoint molecules on the cell surface of T-cells of AML patients has not yet been analyzed, although the combination of flt-3 inhibition with checkpoint inhibitors is currently being tested in Phase I to III trials in this disease.

The Bruton’s tryosine kinase (BTK) inhibitor ibrutinib binds covalently to BTK, thus inhibiting B-cell-receptor-mediated proliferation, inducing apoptosis and migration of neoplastic B-cells out of the protective micromilieu of lymph nodes. The drug has shown impressive efficacy in CLL [88, 89], particularly in patients with p53 mutations or deletions. Moreover, ibrutinib also binds to and inhibits interleukin-2-inducible T-cell kinase (ITK), thereby leading to a T-helper (Th) cell 1 polarization in vitro and in vivo, which aids in inducing an anti-tumor immune response [90]. When mice carrying aggressive lymphomas, breast or colon cancers, which all were insensitive towards ibrutinib treatment, were treated with anti-PDL1 MAb or a combination of anti-PDL1 MAb and ibrutinib, the combination showed significantly enhanced efficacy over anti-PDL1 mAb monotherapy [91]. In addition, murine and human myeloid-derived suppressor cells, which play a relevant role in suppressing an efficient anti-tumor immune reaction, express BTK and ibrutinib has been shown to eliminate these cells in vivo [92].

In addition, PDL1-exposed lymphocytes cocultured with melanoma cell lines showed downregulation of MAPK signaling which could be reverted by the B-Raf inhibitor vemurafenib in vitro [93]. In murine (transplantation) models for hepatocellular cancer, tumor shrinkage was induced by sorafenib which was linked to a downregulation of PD1+/CD8+ and Treg cells in the tumor microenvironment [94]*.* In addition, in murine B-Raf wild-type syngeneic transplantable tumors Raf-kinase inhibitors paradoxically induced hyperactivation of extracellular-signal regulated kinase (ERK) signaling and thus increased T-cell activation and signaling [95]. This may serve as an explanation for increased anti-tumor activity of the combination of CTLA-4-and Raf-kinase inhibitors in preclinical models. Little has been done to systematically analyze these interactions of Raf-kinase with checkpoint inhibitors on a broader, preclinical level.

Other kinase inhibitors have been shown to increase tumor cell infiltration by T-cells, as detected in core biopsies of patients, which predicts a more favorable spontaneous clinical course and better response to neoadjuvant Her2-targeting agents in breast cancer [96, 97]. These effects predominantly seem to be reflected by the CD8+/Treg ratio within the tumor tissue [98].

Likewise, janus kinase 2 (Jak2) mRNA expression in breast cancer cells was associated with increased numbers of tumor infiltrating leukocytes and better prognosis [99]. However, Jak2-inhibitors, which aim to suppress the growth supporting function of this kinase in tumor cells, are currently tested in clinical trials, but since the detailed role of Jak2 inhibitors on T-cell activation, exhaustion and tumor recognition has not yet been fully addressed, a potential unfavorable effect of Jak2-inhibitors cannot be excluded [99]*.*

### Combination of cytotoxic drugs and checkpoint inhibitors – novel aspects

It is clear that the current results achieved with checkpoint inhibitors in clinical practice are exciting, but far from being good enough. Therefore, various combinations with chemotherapy, radiotherapy or endocrine therapy are currently being tested in clinical trials. This approach was initially followed only reluctantly due to the widespread view that these chemotherapeutic drugs suppress the immune system [100]. However, it is becoming increasingly clear that conventional chemotherapeutics may induce the expression of neoantigens, induce Th1-differentiation and/or suppress Tregs. These drugs have thus been termed ‘immunogenic chemotherapy’ [101], and may ultimately sensitize tumor cells to checkpoint inhibitors [101–104].

In line with this hypothesis, it was demonstrated in a systemically progressive melanoma patient that local radioation therapy induced upregulation of the tumor antigen NY-ESO-1 and resulted in consecutive systemic resensitization towards ipilimumab [105]. This observation was later confirmed in a larger number of patients [106].

Other drugs such as hypomethylating agents may also show additive immunomodulatory effects with checkpoint inhibitors, *via* upregulation of MHC-I on the neoplastic myeloid-derived suppressor cells (for review see [107]). In this regard, the combination of anti-PD1 with anti-CTLA-4 antibodies and 5-azacytidine as well as a histon-deacetylase inhibitor completely eradicated murine breast and colorectal cancer cells in vivo [107].

## The influence of the gut microbiota on the immune response

### Gut microbiota, their development during treatment with chemotherapy and immunomodulators and their influence on the effect of checkpoint inhibitors

Gut microbiota comprise several trillions of microorganisms with a weight of 2 kg (reviewed in [108]). These microorganisms include bacteria, archaea, eukarya and viruses, with the main phyla being firmicutes, bacteroidetes and actinobacteria [108]. Of note, significant interindividual differences in the species and functional composition of the human enterotypes may result from long-term dietary habits [109, 110]. More importantly, gut colonization essentially influences the development of the immune system [111, 112], as seen in inoculation experiments using germ-free mouse models [113–115], and gut microbiota have been reported to be centrally involved in carcinogenesis [116, 117], *eg* in colorectal cancer (for reviews see [118, 119]). Alterations in the composition of gut microbiota have also been shown to exert systemic effects by modulating estrogen metabolism, thereby affecting women’s risk of developing postmenopausal estrogen receptor-positive breast cancer (reviewed in [120]). In addition, certain gut microbiota can induce DNA double-strand breaks and thus adversely influence the genomic stability of intestinal epithelial cells in vitro (eukaryotic cell lines) [121] and in vivo (mouse model) [122]. In addition, gut microbiota may also exert an influence on epigenetic modifications, and can thus influence inflammatory and immunological reactions (reviewed in [108]), and also directly modulate endogenous T-cell immune responses in mice [123].

Gut microbiota also seem to be involved in the regulation of extrathymic differentiation of Tregs in vitro and in vivo [124] and Th1 infiltration into cancer tissues following treatment with cyclophosphamide. Antibiotic treatment –by subsequent changes in gut microbiota– may turn down the effect of immunostimulation exerted by these drugs. In turn, microbiotic constitution within the gut has been shown to be modified in number and class distribution by cytotoxic drugs, including irinotecan, 5-fluorouracil (reviewed in [125]), and others (reviewed in [108]). Vice versa, the microbiota may also be indispensible for the in vivo anti-tumoral effects of certain cytotoxic drugs such as cyclophosphamide [126] or platinum salts [127] as observed in mouse models*.* For example, gut microbiota have been shown to be involved in the metabolization of cytotoxic drugs (reviewed in [108]) and in modifying local toxicity of anticancer drugs in vivo [128–130].

Recently it was also reported that PD1^−/−^ mice have altered composition of the gut microbiota [131], and that the efficacy of anti-CTLA-4 treatment in animals and patients with metastatic melanoma and NSCLC may depend on the constitution of gut bacteria [132]. Studying the interactions between gut microbiota and (i) the efficacy of conventional cytotoxic anticancer drugs, and (ii) immune cells capable of targeting the tumor, are expected to increase our understanding of how one might best therapeutically modulate antitumor immune responses.

## Conclusion

In summary, despite the clinical benefit observed in a relevant proportion of patients by targeted immune checkpoint modulation, this field of research is still in its infancy. However, our increasing understanding of tumor immunology in general, and the immunoediting process exerted by cancer cells in particular, opens a wide window of opportunities to improve therapeutic immunomodulatory approaches against cancer, making translational science in this exciting field more important than ever.

## Burning questions for translational research

- Which factors could serve as predictors of response to checkpoint mediators?
- At which time points and from which source(s) (*ie* peripheral blood or tissue biopsy) should the analysis of potential predictors/biomarkers be performed?
- Which combinations of checkpoint mediators with other therapies seem promising?
- Are the best effects of checkpoint mediators achieved using combinational or sequential approaches?
- What are the optimal time points for which type of immunologic intervention(s)?
- Which biological interactions with the tumor microenvironment might affect the response to checkpoint mediators?

## Abbreviations

A3B: APOBEC3B
AID: Activation-induced cytidine deaminase
AML: Acute myeloid leukemia
APOBEC: Apolipoprotein B mRNA editing enzyme catalytic polypeptide-like
BTK: Bruton’s tryosine kinase
BTLA: B- and T-lymphocyte attenuator
CLL: Chronic lymphocytic leukemia
ctDNA: Circulating tumor DNA
CTLA-4: Cytotoxic T-lymphocyte-associated protein 4
CTLs: Cytotoxic T-cells
EGFR: Epidermal growth factor receptor
ERK: Extracellular-signal regulated kinase
GITR: Glucocorticoid-induced TNFR family related gene
HVEM: Herpesvirus entry mediator
ICOS: Inducible T-cell costimulator
ILA: Induced by lymphocyte activation
ITK: Interleukin-2-inducible T-cell kinase
Jak2: Janus kinase 2
LAG3: Lymphocyte activation gene 3
MAb: Monoclonal antibody/antibodies
MAPK: Mitogen-activated protein kinase
MEK: Mitogen-activated protein kinase kinase
MHC: Major histocomaptibility complex
NSCLC: Non-small-cell lung cancer
OS: Overall survival
PD1: Programmed death 1
PDL1: PD1 ligand
TAA: Tumor-associated antigens
TCR: T-cell receptor
Th: T-helper
TIGIT: T-cell immunoreceptor with Ig and ITIM domains
TIM3: T-cell immunoglobulin and mucin protein 3
TNFR: Tumor necrosis factor receptor
Tregs: Regulatory T-cells
TREML-2: Trem-like transcript 2 protein
TSA: Tumor-specific (neo)antigens
VISTA: V-domain Ig suppressor of T-cell activation
